# Recruitment of Host Nuclear Pore Components to the Vicinity of *Theileria* Schizonts

**DOI:** 10.1128/mSphere.00709-19

**Published:** 2020-02-05

**Authors:** Sandra Huber, Anina Bär, Selina Epp, Jacqueline Schmuckli-Maurer, Naja Eberhard, Bruno M. Humbel, Andrew Hemphill, Kerry Woods

**Affiliations:** a Institute for Animal Pathology, Vetsuisse Faculty, University of Bern, Bern, Switzerland; b Institute of Cell Biology, University of Bern, Bern, Switzerland; c Institute for Parasitology, Vetsuisse Faculty, University of Bern, Bern, Switzerland; d Electron Microscopy Facility, University of Lausanne, Lausanne, Switzerland; Johns Hopkins Bloomberg School of Public Health

**Keywords:** annulate lamellae, apicomplexan, importin, nuclear pore complex, *Theileria*

## Abstract

Parasitic protozoans of the genus *Theileria* are intracellular pathogens that induce the cellular transformation of leukocytes, causing uncontrolled proliferation of the infected host cell. The transforming stage of the parasite has a strictly intracellular lifestyle and ensures its distribution to both daughter cells during host cell cytokinesis by aligning itself across the metaphase plate and by binding tightly to central spindle and astral microtubules. Given the importance of the parasite surface in maintaining interactions with host microtubules, we analyzed the ultrastructure of the host-parasite interface using transmission electron microscopy combined with high-resolution fluorescence microscopy and live-cell imaging. We show that porous membranes, termed annulate lamellae (AL), closely associate with the *Theileria* surface in infected T cells, B cells, and macrophages and are not detectable in noninfected bovine cell lines such as BL20 or BoMACs. AL are membranous structures found in the cytoplasm of fast-proliferating cells such as cancer cells, oocytes, and embryonic cells. Although AL were first observed more than 60 years ago, the function of these organelles is still not known. Indirect immunofluorescence analysis with a pan-nuclear pore complex antibody, combined with overexpression of a panel of nuclear pore proteins, revealed that the parasite recruits nuclear pore complex components close to its surface. Importantly, we show that, in addition to structural components of the nuclear pore complex, nuclear trafficking machinery, including importin beta 1, RanGAP1, and the small GTPase Ran, also accumulated close to the parasite surface.

**IMPORTANCE** *Theileria* schizonts are the only known eukaryotic organisms capable of transforming another eukaryotic cell; as such, probing of the interactions that occur at the host-parasite interface is likely to lead to novel insights into the cell biology underlying leukocyte proliferation and transformation. Little is known about how the parasite communicates with its host or by what route secreted parasite proteins are translocated into the host, and we propose that nuclear trafficking machinery at the parasite surface might play a role in this. The function of AL remains completely unknown, and our work provides a basis for further investigation into the contribution that these porous, cytomembranous structures might make to the survival of fast-growing transformed cells.

## Abstract (precis)

*Theileria* schizonts are the only known eukaryotic organisms capable of transforming another eukaryotic cell; as such, probing of the interactions that occur at the host-parasite interface is likely to lead to novel insights into the cell biology underlying leukocyte proliferation and transformation. Little is known about how the parasite communicates with its host or by what route secreted parasite proteins are translocated into the host, and we propose that nuclear trafficking machinery at the parasite surface might play a role in this. The function of AL remains completely unknown, and our work provides a basis for further investigation into the contribution that these porous, cytomembranous structures might make to the survival of fast-growing transformed cells.

## INTRODUCTION

*Theileria* spp. are intracellular parasites that reside in the cytoplasm of leukocytes. These unique pathogens interact with their host cell in a remarkable manner, rewiring signaling pathways and altering gene expression to such an extent that infected cells become transformed and acquire many features of cancer cells. *Theileria*-transformed cells proliferate rapidly without a dependency on exogenous growth factors, acquire invasive and metastatic properties, and become immortalized (1) (reviewed in references 2, 3, 4, and 5). *Theileria* sporozoites are transmitted via ticks and infect bovine leukocytes by a process of passive endocytosis (6). Soon after invasion of a leukocyte, the surrounding host-derived vacuole is lysed, a process that is essential for the establishment of infection and that allows *Theileria* to avoid lysosomal destruction (7). The parasite rapidly forms a close association with host microtubules (MTs) and undergoes schizogony to become a multinucleated schizont that resides in a free state in the cytoplasm (8). This is in contrast to other apicomplexan parasites such as *Plasmodium* and *Toxoplasma*, which establish their niche within a host membrane-derived parasitophorous vacuole (PV).

The schizont is the life cycle stage responsible for cellular transformation, inducing clonal expansion of infected leukocytes and ultimately causing leukoproliferative diseases in cattle called East Coast fever and tropical theileriosis (reviewed in reference 3). Extensive changes in host kinase signaling pathways and transcription factor activation occur in response to *Theileria* infection, although little is known about the mechanisms by which *Theileria* induces these phenotypic changes (2). While many secreted *Toxoplasma*-encoded effector proteins have been shown to play roles in modulating host gene expression and signaling pathways (9), few *Theileria* effector proteins have been characterized. These include a Theileria annulata peptidyl prolyl isomerase (TaPIN1) that is translocated into the host cell cytoplasm and nucleus, where it activates the oncogenic c-JUN pathway, thus contributing to transformation (10). Other examples include TashAT1, TashAT2, TashAT3, TashHN, and SuAT1, T. annulata proteins that contain mammalian “AT-hook” DNA binding domains and are secreted into the host nucleus (11–14). Considering the cytoplasmic location of the schizont, it has been proposed that the parasite surface could function as a signal transduction platform (4, 15). A striking example that supports this hypothesis is the recruitment of host cell IκB kinase (IKK) signalosomes into active signaling complexes at the parasite membrane. The constitutive activation of IKK complexes leads to sustained activity of NF-κB, which in turn is essential for the survival of T. parva-infected and T. annulata-infected cells (16). Another example is the sequestration of tumor suppressor protein p53 at the T. annulata surface followed by the prevention of nuclear translocation and inhibition of the p53 apoptotic pathway (17). c-Jun-N-terminal kinase 2 (JNK2) associates with the schizont surface via an interaction with T. annulata p104, potentially contributing to both the survival and dissemination of parasitized cells (18). We recently identified a family of host adaptor proteins, including CD2AP, CIN85, and ASAP1, which coat the parasite surface throughout the cell cycle. These proteins contain multiple protein binding motifs and have the potential to bring together large signaling complexes. We showed that CD2AP forms a large complex composed of several parasite surface molecules along with host-encoded microtubule-associated proteins (MAPs), including CLASP1 and EB1 (15). The survival of the transformed host cell and that of the strictly intracellular schizont are intricately linked—one cannot survive without the other. The parasite ensures its persistence within the cytoplasm by inducing the formation of stable MT bundles at its surface and by integrating itself into the central spindle of the host cell during cytokinesis (19, 20), interactions that are mediated at least in part by the recruitment of CLASP1, EB1, and mitotic kinase Plk1 to the parasite surface (20–22).

Currently, the most therapeutically active compound in use for the treatment of East Coast fever and tropical theileriosis is buparvaquone. Buparvaquone targets the electron transport chain of the parasite, and treatment of infected leukocytes leads to a stop in proliferation and the onset of apoptosis of the host cell within 2 to 3 days (23). We recently used quantitative reverse transcriptase real-time PCR and electron microscopy (EM) to study the effects of buparvaquone on parasite gene expression and ultrastructure (24). During the course of those studies, we were intrigued by the observation that electron-dense membranous structures, which we referred to as “button-like structures,” were frequently found in close proximity to the schizont surface (24). A literature search uncovered an ultrastructural study in which these membranous structures were observed in close proximity to the schizont in *Theileria*-transformed leukocytes (25). Those authors noted a morphological similarity to annulate lamellae (AL), which are cytomembranous structures embedded with pores that are structurally and biochemically similar to nuclear pore complexes (NPCs) (26). AL have been identified in a wide range of cell types, most frequently in transformed and embryonic cells. Examples include oocytes of zebrafish and humans (27) as well as several different cancer and tumor cells (28). The only other report of AL in parasitized cells that we are aware of described the occurrence of AL in giant cells of the face fly Musca autumnalis following infection with the nematode *Thelazia* sp. (29). Despite their abundance in a diverse array of cell types, knowledge of the function of AL remains elusive. One of the most important and best-studied functions of NPCs is that of mediating the trafficking of macromolecules between the nucleus and the cytoplasm. While ions and small (<40-kDa) metabolites can diffuse through NPCs, larger macromolecules must be actively transported. Nuclear trafficking depends on karyopherins such as importin and exportin, which recognize cargo molecules containing nuclear localization signals (NLSs) and nuclear export signals (NES), respectively, and on the formation of a Ran GTPase gradient (30). Whether AL pore complexes (ALPCs) are involved in nuclear transport or in the formation of a Ran gradient is not fully clear (31). Until now, no attempts have been made to characterize AL in *Theileria*-transformed cells.

We recently performed a proximity-dependent biotinylation (BioID) analysis of the parasite surface, and we found that several nuclear pore complex proteins (Nups), including RanBP2 and nucleoporin 214 (Nup214), accumulated in the close vicinity of the schizont (15). We therefore decided to perform an in-depth analysis of the host-parasite interface using transmission electron microscopy (TEM) combined with fluorescence microscopy. In particular, we aimed to test the putative association of host cell Nups with porous membranes at the parasite surface. Immunogold labeling confirmed that parasite-associated AL are indeed composed of nuclear pore complex proteins, and fluorescence microscopy and live-cell imaging revealed that not only structural components of NPCs but also various forms of nuclear-cytoplasmic trafficking machinery, including importin and Ran, associate with the parasite surface.

## RESULTS

### Nuclear pore-containing membranes align closely with the *Theileria* schizont surface.

**FIG 1:**
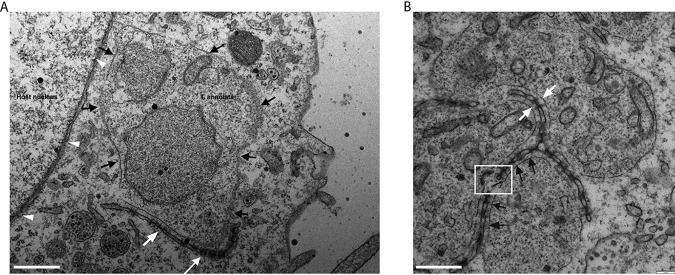
TEM analysis of T. annulata-infected macrophages reveals the presence of porous membranes in close proximity to the schizont surface. Glutaraldehyde-fixed and Epon-embedded TaC12 cells were analyzed using TEM. Parasite associated annulate lamellae are labeled with white arrows, the schizont surface is labeled with black arrows, and nuclear pores are labeled with white arrowheads. (A) T. annulata schizont surrounded by a porous membrane. Note that pores are visualized as spheres or cross-sections, depending on the plane of view. Bar, 380 nm. (B) T. annulata schizont surrounded by a porous membrane. The white box indicates a potential cytostome. Bar, 500 nm.

TEM of a T. annulata-infected macrophage cell line (TaC12) revealed electron-dense membranes that are embedded with pores and closely associate with the surface of the parasite (Fig. 1). These porous membranes were most often found in very close proximity to but not touching the schizont membrane. The pores, which are viewed as spheres or as channels (i.e., as cross-sections), depending on the plane of view, are morphologically indistinguishable from nuclear pores (Fig. 1A, white arrows compared to white arrowheads). These porous structures resemble AL, cytoplasmic organelles consisting of stacked sheets of membranes embedded with NPCs (26). ALPCs are morphologically and biochemically similar to NPCs, although their function remains unknown (31). Structures morphologically similar to AL have previously been observed in both T. annulata-infected and T. parva-infected leukocytes (25). In some cases, fragments of AL were detected within phagocytic vacuoles in the schizont cytosol, leading to the suggestion that the phagocytosis of AL by the parasite occurs via a cytostome. Our observations support this finding, and we frequently detected cytostome-like structures in the schizont membrane situated close to AL (Fig. 1B, white box). We did not, however, detect any internalized AL in this study. TEM analysis of T. annulata-infected macrophages arrested in prometaphase revealed that the association of these membranes with the parasite does not occur during host mitosis. While MTs were clearly seen to align with the parasite surface, we detected no porous membranes (see Fig. S1 in the supplemental material). We could not detect any accumulation of AL in a noninfected bovine cell line (BoMAC), suggesting that the formation of these structures could be linked to the presence of the parasite (data not shown).

10.1128/mSphere.00709-19.1 FIG S1 Nuclear pore-containing membranes are not detected in T. annulata-infected cells during mitosis. T. annulata-infected macrophages were synchronized in a prometaphase-like state by incubating with nocodazole for 16 h. Following repolymerization of microtubules (30 min after nocodazole was washed away), cells were fixed with glutaraldehyde and embedded in Epon for TEM analysis. Host microtubules closely associated with the schizont and are labeled with black arrows. No evidence of porous membranes was found. Bar, 290 nm. Download FIG S1, TIF file, 1.0 MB. Copyright © 2020 Huber et al. 2020 Huber et al. This content is distributed under the terms of the Creative Commons Attribution 4.0 International license.

### The *Theileria* schizont recruits nuclear pore complex proteins in a cell cycle-dependent manner.

We recently reported that some components of the nuclear trafficking machinery, including RanGAP1, RanBP2, Nup214, and Nup160, are localized in close proximity to the parasite surface (15). Considering that the parasite-associated ALPCs observed by TEM are morphologically similar to nuclear pores, we asked whether they are composed of structural nuclear pore proteins. NPCs are large protein complexes consisting of multiple copies of at least 30 different proteins, called nucleoporins (Nups) (reviewed in reference 32). To test whether other Nups can associate with the parasite, we transfected a panel of Nups in *Theileria*-infected cells and analyzed their localization by indirect immunofluorescence using an antinucleoporin monoclonal antibody (MAb), namely, MAb414, which recognizes the FxFG repeat sequence in several nucleoporins, including Nup62, Nup152, and Nup90, and is routinely used as a marker of NPCs (33). These experiments revealed that seven of eight tested nucleoporins localized not only to the nuclear envelope but also in close proximity to the parasite surface (Fig. S2 and data not shown). This was the case for components of the cytoplasmic filaments (Nup214 and RanBP2), the inner pore ring (Nup35 and Nup93), and the nuclear and cytoplasmic outer rings (Nup37 and Nup160). The central channel of NPCs is lined by Nups containing Phe- and Gly-rich repeats (central FG-repeat Nups), and a core member of this group, Nup 62, was also found close to the parasite. Finally, we tested the localization of POM121, a transmembrane nucleoporin that binds to the inner nuclear membrane, and of Nup153, a nuclear basket protein that acts as the docking site for an importing karyopherin (34). While GFP-POM121 (green fluorescent protein-POM121) was localized in close proximity to the parasite surface, GFP-Nup153 was found exclusively on the nuclear envelope. It has been previously reported that proteins of the inner nuclear membrane complex, such as lamin, do not localize to AL (35). We found that *Theileria*-associated AL do not contain laminB1 (data not shown) and are thus similar in protein composition to AL reported in other cell types (36).

10.1128/mSphere.00709-19.2 FIG S2 Host cell nucleoporins localize to the vicinity of the parasite. (A) T. annulata-infected macrophages were transfected with GFP-Nup35, GFP-Nup62, GFP-POM121, and GFP-Nup153 and processed for indirect immunofluorescence analysis. Nup93, Nup37, Nup160, and Nup214 also localize close to the parasite surface (not shown). Nucleoporins were labeled with MAb414, the parasite surface was labeled with anti-TaSP (red), and DNA was labeled with DAPI. A merge of the data representing GFP fusion protein expression and the schizont (green and red) is shown. All nucleoporins tested (except for Nup153) were recruited to the schizont surface. Bar, 10 μm. (B) Schematic showing the location and structural organization of selected Nups in relation to nuclear trafficking proteins (59). All but one of the proteins tested were found to localize in close proximity to the parasite surface (indicated in green). Only Nup153 did not associate with the parasite surface (indicated in red). Nup35 and Nup93 are components of the inner ring complex. POM121 is a transmembrane Nup. Nup37 and Nup160 are components of the outer ring complex (Y-complex). Nup214 and RanBP2 localize to cytoplasmic filaments. Nup62 is a central channel FG repeat-containing Nup. Nup153 is a component of the nuclear basket. Importin beta is a karyopherin that transports proteins into the nucleus by binding to nuclear localization signals. Ran is a small GTPase that exists in a GDP-bound or GTP-bound form and is essential for translocation of RNA and proteins through NPCs. The schematic was made using PowerPoint and Illustrator. Download FIG S2, TIF file, 2.7 MB. Copyright © 2020 Huber et al. 2020 Huber et al. This content is distributed under the terms of the Creative Commons Attribution 4.0 International license.

**FIG 2:**
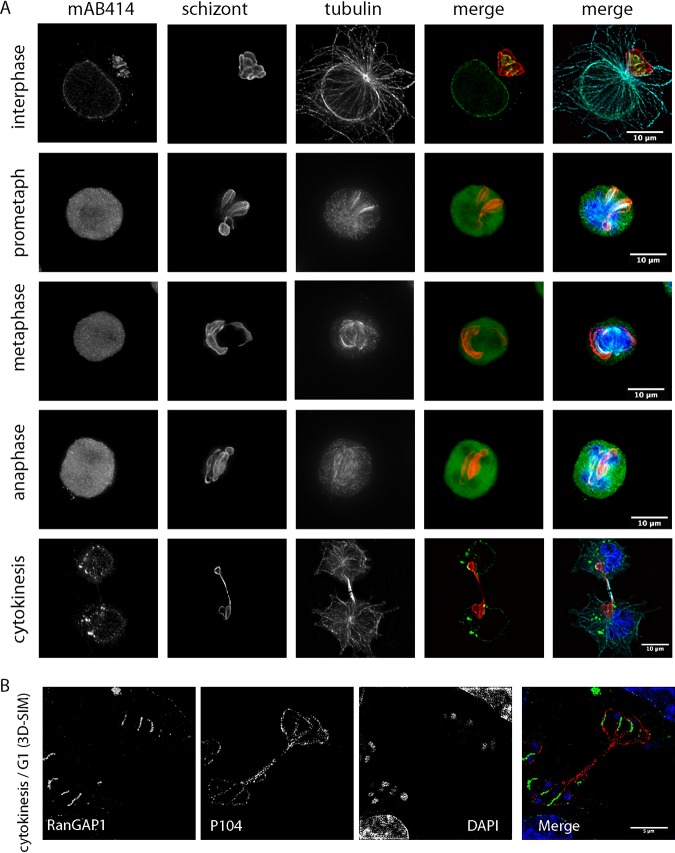
Cell cycle-dependent association of Nups with the schizont surface. (A) Deconvolution images of T. annulata-infected macrophages were taken at different stages of the cell cycle. Cells were fixed and analyzed by indirect immunofluorescence analysis with anti-nucleoporin (MAb414 [green]) antibodies. The parasite was labeled with anti-TaSP (red), microtubules ware labeled with anti-alpha tubulin (cyan) antibodies, and DNA was labeled with DAPI (blue). Representative cells are shown in interphase, prometaphase, metaphase, anaphase, and telophase/cytokinesis. Bar, 10 μm. (B) A T. annulata-infected macrophage in the late stages of cytokinesis/G_1_ phase was imaged using 3D-SIM microscopy (DeltaVision OMX Blaze). RanGAP1 is labeled with green, and the parasite surface was labeled with anti-p104 (1C12) antibodies (red). One z-stack is shown. Bar, 5 μm.

To analyze the localization of Nups during the cell cycle and to support our observations made using overexpressed GFP-fusion proteins, TaC12 cells were synchronized in prometaphase and were fixed at different stages of the cell cycle, prior to processing for immunofluorescence analysis using MAb414. During interphase, when the host nuclear envelope remains intact, we observed that the MAb414 signal, in addition to its expected nuclear envelope localization, accumulates in a distinct pattern along and between the lobes of the schizont membrane (Fig. 2A). This striking pattern was identified in 100% of the interphase cells analyzed (*n* > 500) (Fig. S3A and B). As cells entered mitosis and the nuclear envelope broke down, MAb414 foci were found in a diffuse pattern throughout the cytoplasm, with no association with either host cell DNA or the parasite surface. During cytokinesis, MAb414 foci reassociated with the newly formed nuclear envelope and were additionally detected in cytoplasmic puncta. Finally, as cells completed cytokinesis and entered G_1_ phase, MAb414 foci were again detected at the parasite surface in a distinct pattern that resembled parallel lines, or “finger-like” projections (Fig. 2A). We observed a similar cell cycle-dependent pattern for Ran GTPase activating protein 1 (RanGAP1), suggesting that this dynamic cell cycle-dependent localization also applies to other components of the nuclear trafficking machinery (Fig. S4). Three-dimensional (3D) reconstruction following superresolution deconvolution microscopy revealed that RanGAP1 coats parts of the parasite surface between the lobes of the schizont (Fig. 2B; see also Movie S1 and S2 in the supplemental material). Live-cell imaging of GFP-RanGAP1-expressing TaC12 cells as they progressed through anaphase/telophase suggested that RanGAP1 first associated with the nuclear envelope before accumulating in punctate structures in the cytoplasm. The RanGAP1 signal began to accumulate near the parasite surface only after the nuclear envelope was complete (Movie S3; see also Fig. S5). Analysis of the distribution of MAb414 foci in five different T. annulata-infected macrophage lines, including a clonal cell line infected with T. annulata isolated from Soba, Sudan (a gift from Ivan Morrison), and Thei, a well-established laboratory line derived from a naturally infected animal (37), reassured us that AL formation is not a cell type-specific anomaly in TaC12 cells (Fig. S6A and B). Following 48 h of buparvaquone treatment, MAb414-positive foci were still seen to be associated with the remaining schizont membranes, suggesting that the localization or maintenance of AL in *Theileria*-infected cells does not depend on the presence of a viable parasite (Fig. S6C and D).

10.1128/mSphere.00709-19.3 FIG S3 RanGAP1, MAb414 foci, and importin beta 1 are found at the schizont surface in all interphase cells. T. annulata-infected macrophages were processed for immunofluorescence analysis performed with anti-RanGAP1 and importin beta 1 antibodies (green), and Nups were detected with MAb414 (green). The schizont was labeled with 1C12 monoclonal antibody or polyclonal anti-TaSP antibodies (red), and DNA was labeled with DAPI (blue). Bar, 10 μm. Download FIG S3, TIF file, 1.3 MB. Copyright © 2020 Huber et al. 2020 Huber et al. This content is distributed under the terms of the Creative Commons Attribution 4.0 International license.

10.1128/mSphere.00709-19.4 FIG S4 RanGTPase activating protein 1 (RanGAP1) localizes to the T. annulata schizont surface during interphase. Deconvolution images representing T. annulata-infected macrophages were taken at different stages of mitosis. Cells were fixed and subjected to indirect immunofluorescence analysis with anti-RanGAP1 (cyan) antibodies. The parasite was labeled with anti-p104 (1C12; red), microtubules were labeled with anti-CLASP2 (green) antibodies, and DNA was labeled with DAPI (blue). Representative cells are shown in interphase, metaphase, and telophase/cytokinesis. Bar, 10 μm. Download FIG S4, TIF file, 2.5 MB. Copyright © 2020 Huber et al. 2020 Huber et al. This content is distributed under the terms of the Creative Commons Attribution 4.0 International license.

10.1128/mSphere.00709-19.5 FIG S5 GFP-RanGAP1 reassociates with the schizont surface following mitosis. Representative images from movie S3 are shown. TaC12 cells expressing GFP-RanGAP1 and CLASP1_1256–1538_-mCherry were imaged by time-lapse microscopy, and the images were deconvolved using DeltaVision integrated software. One image was taken every 2 min, and a single z-stack is shown. Bar, 10 μm. Download FIG S5, TIF file, 1.3 MB. Copyright © 2020 Huber et al. 2020 Huber et al. This content is distributed under the terms of the Creative Commons Attribution 4.0 International license.

10.1128/mSphere.00709-19.6 FIG S6 Localization of Nups in T. annulata-infected macrophages (TaSOBA and Thei cells) and following buparvaquone treatment in TaC12 cells. (A and B) T. annulata-infected macrophages (TaSOBA [A] and Thei [B]) were processed for immunofluorescence analysis. Nups were detected with MAb414 (green). The schizont was labeled with polyclonal anti-TaSP antibodies (red), and DNA was labeled with DAPI (blue). Bar, 10 μm. (C and D). TaC12 cells were treated with 150 nM buparvaquone for 48 h prior to processing for immunofluorescence analysis with MAb414 (C) or anti-importin beta 1 (green) (D). The schizont was detected with anti-TaSP antibodies (red), and DNA was labeled with DAPI (blue). Bar, 10 μm. Download FIG S6, TIF file, 0.9 MB. Copyright © 2020 Huber et al. 2020 Huber et al. This content is distributed under the terms of the Creative Commons Attribution 4.0 International license.

10.1128/mSphere.00709-19.7 MOVIE S1 RanGTPase activating protein 1 (RanGAP1) localizes to the T. annulata schizont surface (projection through z-stacks). A T. annulata-infected macrophage in the late stages of cytokinesis/G_1_ phase was imaged using 3D-SIM microscopy (DeltaVision OMX Blaze). RanGAP1 is labeled in green, p104 is labeled in red, DNA was labeled with DAPI (blue). A projection through multiple z-stacks is shown. Bar, 5 μm. Download Movie S1, MOV file, 0.8 MB. Copyright © 2020 Huber et al. 2020 Huber et al. This content is distributed under the terms of the Creative Commons Attribution 4.0 International license.

10.1128/mSphere.00709-19.8 MOVIE S2 RanGTPase activating protein 1 (RanGAP1) localizes to the T. annulata schizont surface (3D volume projection). A T. annulata-infected macrophage in the late stages of cytokinesis/G_1_ phase was imaged using 3D-SIM microscopy (DeltaVision OMX Blaze). RanGAP1 is labeled in green, p104 is labeled in red, DNA was labeled with DAPI (blue). A 3D volume projection is shown. Download Movie S2, MOV file, 1.1 MB. Copyright © 2020 Huber et al. 2020 Huber et al. This content is distributed under the terms of the Creative Commons Attribution 4.0 International license.

10.1128/mSphere.00709-19.9 MOVIE S3 GFP-RanGAP1 reassociates with the schizont surface following mitosis. TaC12 cells expressing GFP-RanGAP1 and CLASP1_1256–1538_-mCherry were imaged by time-lapse microscopy, and images were deconvolved using DeltaVision integrated software. One image was taken every 2 min, and a single z-stack is shown. Bar, 10 μm. Download Movie S3, AVI file, 14.2 MB. Copyright © 2020 Huber et al. 2020 Huber et al. This content is distributed under the terms of the Creative Commons Attribution 4.0 International license.

**FIG 3:**
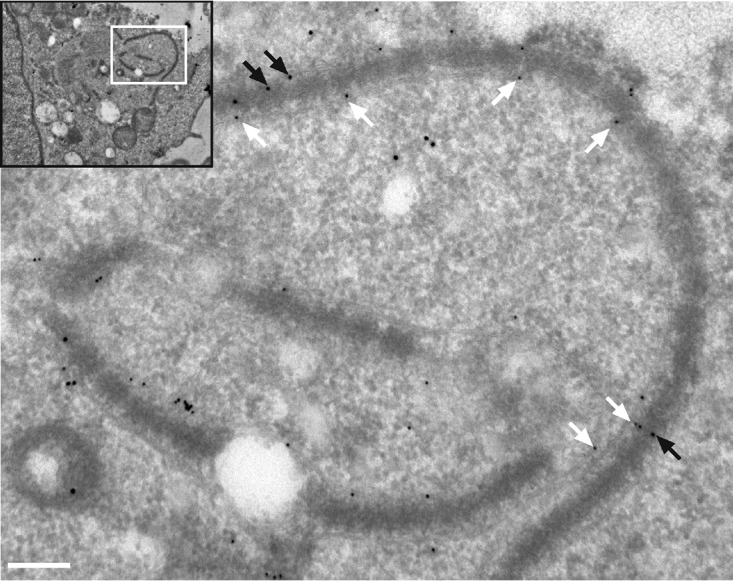
TEM and immunogold labeling with MAb414. T. annulata-infected macrophages were fixed with formaldehyde and processed according to the Tokuyasu technique prior to labeling with rabbit anti-TaSP (10-nm-diameter gold particles; white arrows) and mouse MAb414 (15-nm-diameter gold particles; black arrows). The white box in the inset indicates the magnified area. Bar, 220 nm.

**FIG 4:**
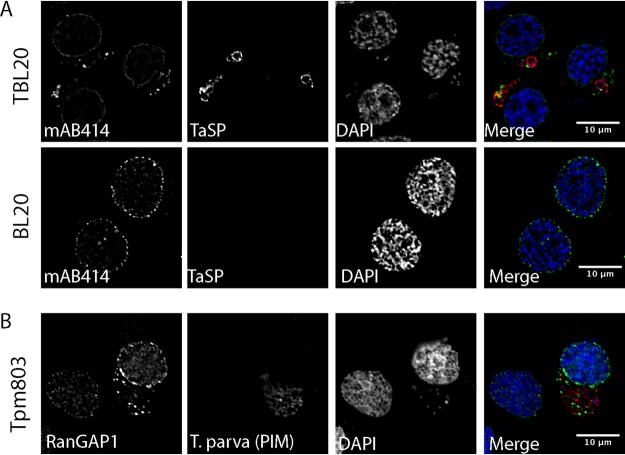
Localization of Nups in T. annulata-infected and T. parva-infected B and T cells. (A) T. annulata-infected bovine B cells (TBL20) and their noninfected counterparts (BL20) were processed for immunofluorescence analysis with MAb414 (green). The parasite was labeled with anti-TaSP (red), and DNA was labeled with DAPI (blue). (B) T. parva-infected T cells (Tpm803) were labeled with anti-RanGAP1 (green). The parasite was labeled with anti-T. parva PIM (red), and DNA was labeled with DAPI (blue). Bar, 10 μm.

**FIG 5:**
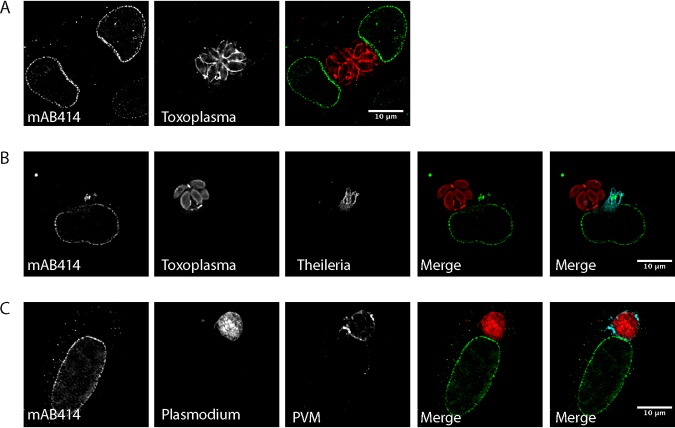
Localization of Nups in Toxoplasma gondii and Plasmodium berghei-infected cells. (A and B) Human foreskin fibroblast (HFF) cells (A) or TaC12 cells (B) were infected with T. gondii and processed for immunofluorescence analysis. Nups are labeled with MAb414 (green). *Toxoplasma* was detected with rabbit anti-*Toxoplasma* serum (red), and *Theileria* was visualized with anti-CLASP1 antibodies (cyan). A merge is shown of MAb414 with *Toxoplasma* (A [green and red]) and MAb414 with *Toxoplasma* and *Theileria* (B [green, red, and cyan]). (C) HeLa cells were infected with mCherry expressing-P. berghei sporozoites and were processed for microscopy 24 h postinfection. Nups were labeled with MAb414 (green), the parasite is indicated in red, and the parasitophorous vacuole (PVM) was detected with anti-UIS4 antibodies (cyan). A merge is shown with MAb414 and *Plasmodium* (green and red) and additionally with the PVM (green, red, and cyan). Bar, 10 μm.

To correlate our observations made by TEM with our fluorescence data, we performed immunogold labeling following the Tokuyasu technique using MAb414 (Fig. 3). The detection of MAb414 signal on electron-dense structures close to the parasite indicated that the parasite-associated AL do indeed contain nuclear pore complexes. AL have been observed in other cell types, particularly in fast-proliferating cells (reviewed in reference 26). To determine whether the AL detected in TaC12 cells represent a characteristic of *Theileria* infection or instead represent a consequence of the proliferative phenotype of *Theileria-*transformed cells, we compared the localizations of Nups in noninfected bovine leukovirus (BLV)-transformed bovine B cells (BL20) and in their *Theileria*-infected counterpart, TBL20 (38). These cells share the same genetic background, and both are fast-growing suspension cell lines. In noninfected BL20 cells, RanGAP1 (not shown) and MAb414 foci (Fig. 4A, bottom panel) were restricted almost exclusively to the nuclear envelope, with only few dispersed clusters of Nups in the cytoplasm. In contrast, MAb414-positive foci were consistently found associating closely with the schizont surface in T. annulata-infected TBL20 cells (Fig. 4A, top panel). Next, we analyzed T. parva-infected T cells (Tpm803) and found that, again, Nups (labeled with RanGAP1) accumulated in the cytoplasm in close proximity to the schizont. In adherent T. annulata transformed cell lines such as TaC12, every cell in culture contains a parasite. In contrast, in cultures of T. parva-infected T cells, cells containing no parasite can be identified in approximately 5% of the culture (16). While MAb414-positive foci (not shown) and RanGAP1-positive foci were frequently seen in close proximity to the T. parva schizont, few cytoplasmic accumulations could be seen in noninfected cells (Fig. 4B), providing further evidence that these structures are induced by the presence of a viable parasite. We consistently noted that AL structures appeared rather extensively punctate in suspension cell lines such as TBL20 and Tpm803 compared to those detected in adherent T. annulata-infected cells. Although cytospin procedure was carried out at low speed as previously described (39), we cannot exclude the possibility that this represents an artifact induced by cytospin processing. However, it seems more likely to us that this is a feature of infected T and B cells. To see whether the redistribution of Nups to the cytoplasm would also be induced by other apicomplexan parasites, we infected human foreskin fibroblast (HFF) cells with Toxoplasma gondii. In HFF cells, MAb414 staining was detected on the nuclear envelope, with some signal dispersed in the cytoplasm. No association of MAb414 foci with T. gondii was observed (Fig. 5A). Similarly, infection of *Theileria*-infected macrophages (TaC12) with T. gondii induced no change in Nup distribution (Fig. 5B). Finally, we tested Plasmodium berghei-infected HeLa cells and detected no parasite-associated MAb414 staining (Fig. 5C) or RanGAP1 foci (not shown). Together, these data suggest that the presence of *Theileria* in the cytoplasm induces the formation of cytoplasmic AL in close proximity to the schizont surface.

### Host nuclear-cytoplasmic trafficking machinery localizes to the parasite surface.

**FIG 6:**
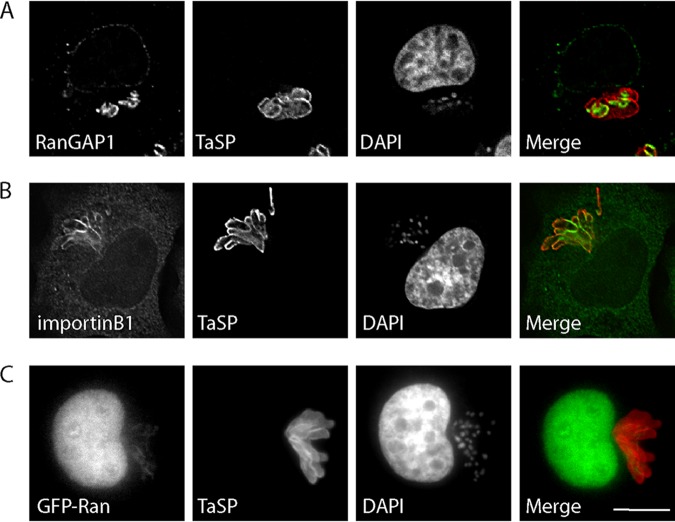
Components of the host cell nuclear transport machinery are recruited to the schizont surface. (A and B) T. annulata-infected macrophages were fixed and analyzed by indirect immunofluorescence analysis with anti-RanGAP1 (A) or anti-importin beta 1 (B) antibodies (green). The parasite was labeled with anti-TaSP (red), and DNA was labeled with DAPI (blue). Bar, 10 μm. (C) TaC12 cells were transfected with GFP-Ran and processed for indirect immunofluorescence analysis. Nucleoporins were labeled with MAb414, the parasite surface was labeled with anti-TaSP (red), and DNA was labeled with DAPI. A merge of GFP-Ran and the schizont (green and red) is shown. Bar, 10 μm.

**FIG 7:**
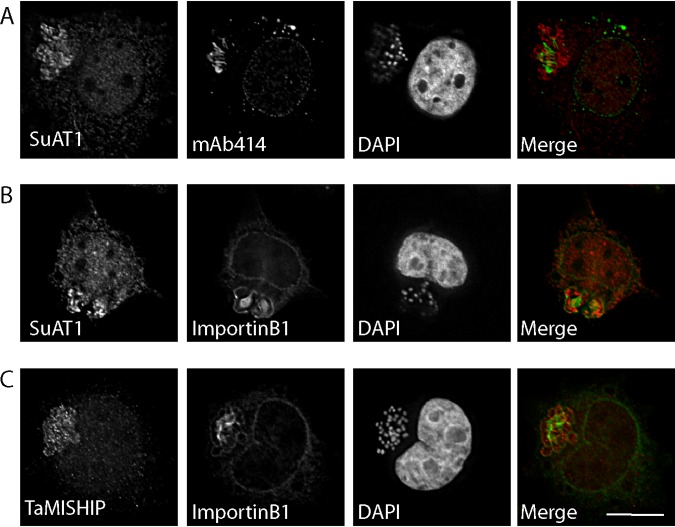
Secreted *Theileria* proteins SuAT1 and TaMISHIP did not colocalize with Nups or importin beta 1 at the schizont surface. T. annulata-infected macrophages were fixed and subjected to indirect immunofluorescence analysis with anti-SuAT1 (A and B) or anti-TaMISHIP (C [red]) together with MAb414 (to label Nups) (A) or anti-importin beta 1 antibodies (green) (B and C). DNA was labeled with DAPI (blue). Bar, 10 μm.

Considering the presence of RanGAP1 close to the parasite (Fig. 2B; see also Fig. 6A) (15), we hypothesized that, in addition to structural components of NPCs, nuclear transport factors, such as Ran and importin, would also accumulate at the schizont surface. Immunofluorescence analysis performed with an anti-importin beta 1 monoclonal antibody and overexpression of GFP-importin beta 1 (not shown) revealed an accumulation of importin beta 1 in the cytoplasm and nucleus of the host cell, at the nuclear envelope, and very clearly along the full length of the parasite surface (Fig. 6B). Unlike the pattern observed for structural components of NPCs (as labeled with MAb414), importin beta 1 appears to accumulate along the full length of the schizont surface. Bovine and *Theileria* Ran are >70% similar at the protein level, and we failed to find an antibody that exclusively detected host cell Ran without a strong signal within the parasite nucleus. Therefore, we transiently transfected GFP-Ran and detected GFP-Ran close to the parasite surface, albeit at much lower intensity than within the host nucleus (Fig. 6C). Live-cell imaging of T. annulata-infected macrophages expressing GFP-Ran confirmed the dynamic association of nuclear trafficking machinery with the parasite (Movie S4). We considered the possibility that parasite-associated nuclear trafficking machinery might be involved in the export or trafficking of secreted *Theileria* proteins, and, to test this, we compared the localizations of T. annulata SuAT1 (12) and TaMISHIP (15) with bovine Nups and importin beta 1. Although both *Theileria* proteins were detected at the surface of the parasite as well as (in the case of SuAT1) in the nucleus, no convincing colocalization with MAb414 and anti-importin beta 1 signals was observed (Fig. 7).

10.1128/mSphere.00709-19.10 MOVIE S4 GFP-Ran localizes to the T. annulata schizont surface in a cell cycle-dependent manner. T. annulata-infected macrophages were transfected with GFP-Ran and CLASP1_1256–1538_-mCherry and imaged by time-lapse wide-field fluorescence microscope. One image was taken every 10 min. Bar, 10 μm. Download Movie S4, MOV file, 5.0 MB. Copyright © 2020 Huber et al. 2020 Huber et al. This content is distributed under the terms of the Creative Commons Attribution 4.0 International license.

## DISCUSSION

By combining TEM with immunogold labeling, high-resolution fluorescence microscopy, and live-cell imaging, we have shown that porous host cell-derived membranes associate closely with the *Theileria* schizont surface. Our work supports earlier observations made using TEM (24, 25) and demonstrates for the first time that these membranes, termed AL, contain NPC components. ALPCs are structurally indistinguishable from NPCs and have been reported to contain most of the nucleoporins tested (36, 40, 41). We tested representative components of all the main structural parts of NPCs for association with AL in *Theileria*-infected cells and found that all except for Nup153, a scaffold element in the nuclear phase, colocalized with AL when overexpressed. We also show that AL are present in *Theileria*-infected cells in a cell cycle-dependent manner, with their dynamics correlating with that of the nuclear envelope. Importantly, we have shown that not only structural components of NPCs accumulate close to the parasite but also functional mediators of nucleocytoplasmic transport, including importin beta 1 and the small GTPase Ran.

AL have been detected in many different cell types, although knowledge of their functional significance remains elusive. AL are often abundant in fast-proliferating cells such as tumor cells, embryonic cells, and oocytes (26, 42). One characteristic feature of *Theileria* infection is the onset of uncontrolled proliferation (43), and we wished to exclude the possibility that schizont-associated AL would be present simply as a consequence of their high rate of proliferation. To test this, we compared the levels of AL distribution in the bovine leukosis cell line (BL20) and in the corresponding infected counterpart (TBL20). Both cell lines proliferate rapidly and continually in culture, although significant parasite-dependent differences in gene expression and metabolism have been reported for these cell lines (44–46). Almost no MAb414 foci outside the nuclear envelope were detectable in BL20, while the characteristic AL aligning with the schizont membranes were present in TBL20, as well as in T. parva-infected T cells. This suggests that it is the presence of the parasite in the cytoplasm that induces the formation of these striking AL in bovine leukocytes. It would be interesting to check for the presence of AL in the cytoplasm of cells infected with nontransforming *Theileria* species, such as T. orientalis. The life cycle of T. orientalis is similar to that of other *Theileria* species, although schizonts are detectable for only a very limited time and do not induce transformation and leukoproliferation and no schizont cell culture system exists (reviewed in reference 47) (Kyoko Hayashida, personal communication). One hypothesis that has been proposed is that AL could function as a storage site for Nups during phases of rapid cell proliferation (26), although this seems an insufficient explanation because, in our hands, BL20 cells proliferated at a rate similar to that seen with TBL20 (data not shown). Further, studies in *Drosophila* early embryos have indicated that AL in fact play only a minor role in Nup storage, because the number of ALPCs did not decrease as the levels of NPCs increased (48). One remarkable feature of *Theileria* infection is the coordination between parasite DNA replication and the host cell cycle. Parasite DNA synthesis has been shown to occur when host cells are in mitosis (49), although how the parasite senses the cell cycle stage of the host remains unknown. In the present study, we detected AL localizing close to an apparent cytostome of the parasite, and others have detected AL internalized within phagosomes of the parasite (25). One attractive hypothesis that merits further investigation is that the schizont can monitor changes in host cell cycle status through the internalization of host-derived AL.

The results seen with *Theileria*-associated AL reminded us of rearrangements of host cell membranes that are induced by various viruses. For example, hepatitis C virus (HCV) induces the formation of a membrane structure termed a “membranous web.” This consists of double-membrane vesicles, is derived from the endoplasmic reticulum (ER), and comprises a virus-induced separate compartment within the cytoplasm (50). One proposed function of the membranous web is in the spatial concentration of viral components, as well as in hiding viral RNA from pattern recognition receptors (PRRs). Multiple Nups and karyopherins were found to accumulate in the membranous web at sites of HCV replication or assembly, and nuclear transport substrates that are normally targeted to the nucleus enter regions of the membranous web. The targeting of NLS-containing proteins to the vicinity of the virus within the membranous web was confirmed by expressing the product of a chimeric gene encoding two tandemly repeated GFP proteins fused to a canonical simian virus 40 (SV40) NLS. This fusion protein accumulated in regions of the membranous web, as well as in the nucleus, when overexpressed in HCV-infected cells (50). Others have shown that NLSs of the basic and M9 types, but not nuclear export signals (NES), direct proteins to AL in *Xenopus* oocytes, providing evidence that AL share functional binding properties with the NPC and that cytoplasmic AL can provide a site for accumulation of nuclear proteins (51). We therefore tested if schizont-associated pores could be playing a similar role, hypothesizing that the schizont might hijack Nups in order to target NLS-containing proteins to the schizont surface. We hypothesized that this could be a strategy employed by the parasite to prevent the nuclear translocation of certain proteins, such as p53, whose nuclear translocation is prevented by sequestration at the parasite surface (17). Though the presence of endogenous p53 has been demonstrated at the surface of the T. annulata schizont (17, 52), we found no convincing colocalization between p53 and AL labeled with RanGAP1 near the parasite surface (data not shown). We found that overexpressed GFP-p53 was found exclusively in the nucleus of TaC12 cells, preventing us from testing this hypothesis by mutation of the NLS. We also found that fusion of GFP to the SV40 canonical NLS in *Theileria*-infected cells resulted exclusively in nuclear accumulation, with nothing detected in the vicinity of the parasite, and a chimeric protein consisting of repeated GFP proteins fused to the NLS of TaMISHIP, a parasite protein that we previously showed to localize to the host nucleus when overexpressed (15), also failed to localize to the schizont (data not shown). Thus, our data do not support the hypothesis that schizont-associated AL function to bind nuclear transport cargo. However, there are many NLSs that do not match the consensus sequence of classical NLSs (53), and so we cannot entirely exclude this hypothesis. *Theileria* schizonts in the host cytoplasm dramatically alter the gene expression and phenotype characteristics of their host cell, and one way in which this is likely to be mediated is via the translocation of secreted parasite proteins to the host nucleus. The route by which such secreted proteins are trafficked from the parasite to the host nucleus remains unclear, and we considered the hypothesis that karyopherins such as importin beta 1, found to accumulate near the parasite surface, could play a key role in host-parasite protein trafficking. Although no striking colocalization of T. annulata SuAT1 and TaMISHIP with bovine NPCs or importin beta 1 was observed, these data do not exclude a possible involvement of ALPCs and/or bovine importin in parasite protein export or trafficking. Indeed, any potential interaction or colocalization between secreted proteins and the ALPC trafficking machinery would be expected to be somewhat transient in nature, and colocalization or interaction is not likely to be frequently detected. Very few *Theileria* proteins have been confirmed as being exported to the host, and, due to lack of available antibodies, in this study we tested the localization of only two candidates. It will be interesting to compare the localizations of as-yet-undiscovered secreted schizont proteins with importin and ALPCs.

## MATERIALS AND METHODS

### Cell culture, parasite propagation.

T. annulata-infected bovine macrophages (TaC12) were cultured at 37°C in Leibovitz 15 medium (Gibco), while TBL20, BL20, Thei, TaSoBA, and Tpm803 cells were cultured at 37°C and 5% CO_2_, in RPMI media, both supplemented with 10% fetal calf serum (FCS; BioConcept), 10 mM HEPES (Merck) (pH 7.2), 2 mM l-glutamine (Lonza), 70 μM β-mercaptoethanol (Merck), and antibiotics (Lonza). Human foreskin fibroblast (HFF) cells were maintained in Dulbecco’s modified Eagle medium (DMEM) containing 10% FCS and antibiotics, and HeLa cells were cultured in minimum essential medium with Earle’s salts (MEM EBS; 1-31F01-1, Bioconcept) supplemented with 2 mM l-glutamine (Lonza), 10% FCS, and antibiotics. Toxoplasma gondii (ME49, type II strain) parasites were maintained in and isolated from Vero cells and used to infect HFF cells as previously described (54). Plasmodium berghei ANKA parasites, which constitutively express mCherry in the parasite cytosol (*Pb*mCherry) (55), were used to infect HeLa cells. For infection of HeLa cells, salivary glands of P. berghei-infected Anopheles stephensi mosquitoes were isolated and disrupted to release sporozoites as previously described (55, 56). Buparvaquone (23) was provided by Cross Vetpharm Group Limited (Dublin, Ireland) and was kept as 1.5 mM stock solution mixed in dimethyl sulfoxide (DMSO) at −20°C. TaC12 cells were treated with 150 nM buparvaquone for 48 h to kill the parasite (24).

### Transfections, lentiviral transduction, cell cycle synchronization.

Amaxa 4D Nucleofector (Lonza) was used with SF cell line solution and the program DS103 to transfect TaC12 cells. Lentiviral particles were produced and used for transduction as described previously (21). Briefly, HEK293T cells were transfected with a third-generation lentiviral transfer vector (pRRL-RSrII) containing the gene of interest, a second-generation packaging vector (psPAX2), and a vesicular stomatitis virus G (VSV-G) coat envelope vector (pMD2.G) by the use of FuGENE HD transfection reagent (Roche). Lentiviral particles were harvested 48 h and 72 h after transfection and were used for three subsequent transductions of TaC12 cells. Cell cycle synchronization was achieved by depolymerization of microtubules with 0.1 μg/ml nocodazole (Biotrend Chemicals) for 16 h. Mitotic cells were harvested by shaking and were released into nocodazole-free medium for up to 2 h (20).

### Expression constructs.

The following expression constructs were purchased from Euroscarf: pEGFP-Nup214, POM121-EGFP3, Nup62-EGFP3, pEGFP-Nup35, pEGFP-Nup160, pEGFP-Nup37, Nup93-EGFP3, pEGFP3-Nup153, and pEGFP-importin B1. pEGFP-RanGAP1 and GFP-p53 were purchased from Addgene (catalog no. 13378 and 12091). Bovine Ran was amplified from TaC12 cDNA and cloned into the XhoI and SalI restriction sites of the pRRL-RsrII plasmid (addgene 12252) for production of lentiviruses. To label the parasite surface for live-cell imaging, CLASP_1256–1538_ was amplified and cloned with a C-terminal mCherry tag into the RsrII restriction site of the pRRL-RsrII plasmid.

### Antibodies.

The following antibodies were used: MAb414 (ab24609, Abcam) (1:500 dilution), anti-RanGAP1 (ab92369, Abcam) (1:500), rabbit anti-lamin B1 (ab16048, Abcam) (1:500 dilution), anti-importin B1 (KPNB1) clone 3E9 (Abcam, ab2811) (1:500), anti-alpha tubulin (clone DM1A; T9026, Sigma) (1:5000), anti-CLASP2 (KT69, Absea) (1:100), anti-TaSP (a gift from Jabbar Ahmed [57]) (1:50,000), mouse monoclonal 1C12 (38) (1:500), rat anti-TaMISHIP (1:500) (15), rabbit anti-SuAT1 (a gift from Brian Shiels [12] [polyclonal R685]) (1:500), and mouse anti-PIM40.3 (ILRI Nairobi) (1:1,000). T. gondii was labeled with whole T. gondii extract rabbit antiserum (54) (1:1,000). The P. berghei parasitophorous vacuole membrane was labeled with rabbit anti-UIS4 antiserum (provided by P. Sinnis, Baltimore, MD, USA) (1:500).

### Immunofluorescence analysis and time-lapse imaging.

TaC12 cells, T. gondii-infected HFF cells, and P. berghei-infected HeLa cells were grown on coverslips prior to fixation with 4% paraformaldehyde for 10 min at room temperature and were permeabilized by incubation in 0.2% Triton X-100 and blocking in 10% heat-inactivated FCS–phosphate-buffered saline (PBS). For analysis of TBL20, BL20, and Tpm803 cells, cytospins were prepared and fixed as described above. Antibodies were diluted in blocking solution, and DNA was stained with DAPI (4′,6-diamidino-2-phenylindole; Invitrogen). The coverslips were mounted using mounting medium (Dako). Samples were analyzed on a DeltaVision Elite high-resolution microscope system (GE Healthcare) with an Olympus IX-70 inverted microscope with a complementary metal oxide semiconductor (CMOS) camera, a 60× Olympus objective, and SoftWorx (Applied Precision) software. The samples shown in Fig. 2B (see also Movie S1 and S2 in the supplemental material) were analyzed on a DeltaVision OMX Blaze system (GE Healthcare) with scientific complementary metal oxide semiconductor (sCMOS) cameras at the Biozentrum in Basel, Switzerland. Images were processed by using Fiji (ImageJ) software and Photoshop (Adobe). Time-lapse imaging was performed using a DeltaVision Elite high-resolution microscope system (GE Healthcare) (movie S3) or an LSM 5 Duo microscope system (Zeiss) (Movie S4).

### Transmission electron microscopy and immunogold labeling.

TaC12 cells were processed for transmission electron microscopy as described previously (24), and grids were viewed on a Philips 400 transmission electron microscope operating at 60 kV or with a Tecnai 12 transmission electron microscope operating at 120 keV (FEI). TaC12 samples were fixed with 2% formaldehyde for 2 h prior to processing with the Tokuyasu technique for immunogold labeling (58). Nucleoporins were labeled with mouse monoclonal MAb414, diluted 1:2,000, and detected with goat anti-mouse 15-nm-diameter gold particles. The schizont surface was labeled with anti-TaSP antibodies and detected with goat anti-rabbit 10-nm-diameter gold particles.
