# Supplementary figures and images for: Recruitment of Host Nuclear Pore Components to the Vicinity of Theileria Schizonts
Source: mSphere. 2020 Feb 5;5(1):e00709-19. doi: 10.1128/mSphere.00709-19 (PMC7002307; doi:10.1128/mSphere.00709-19)

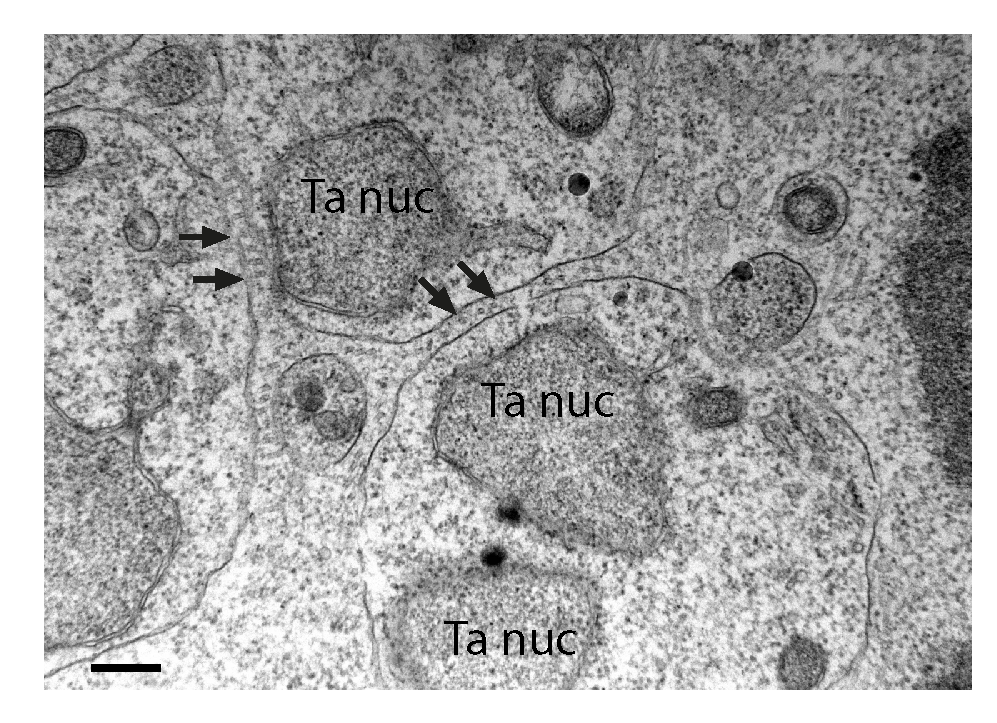

Supplement: FIG S1 [file mSphere.00709-19-sf001.tif]

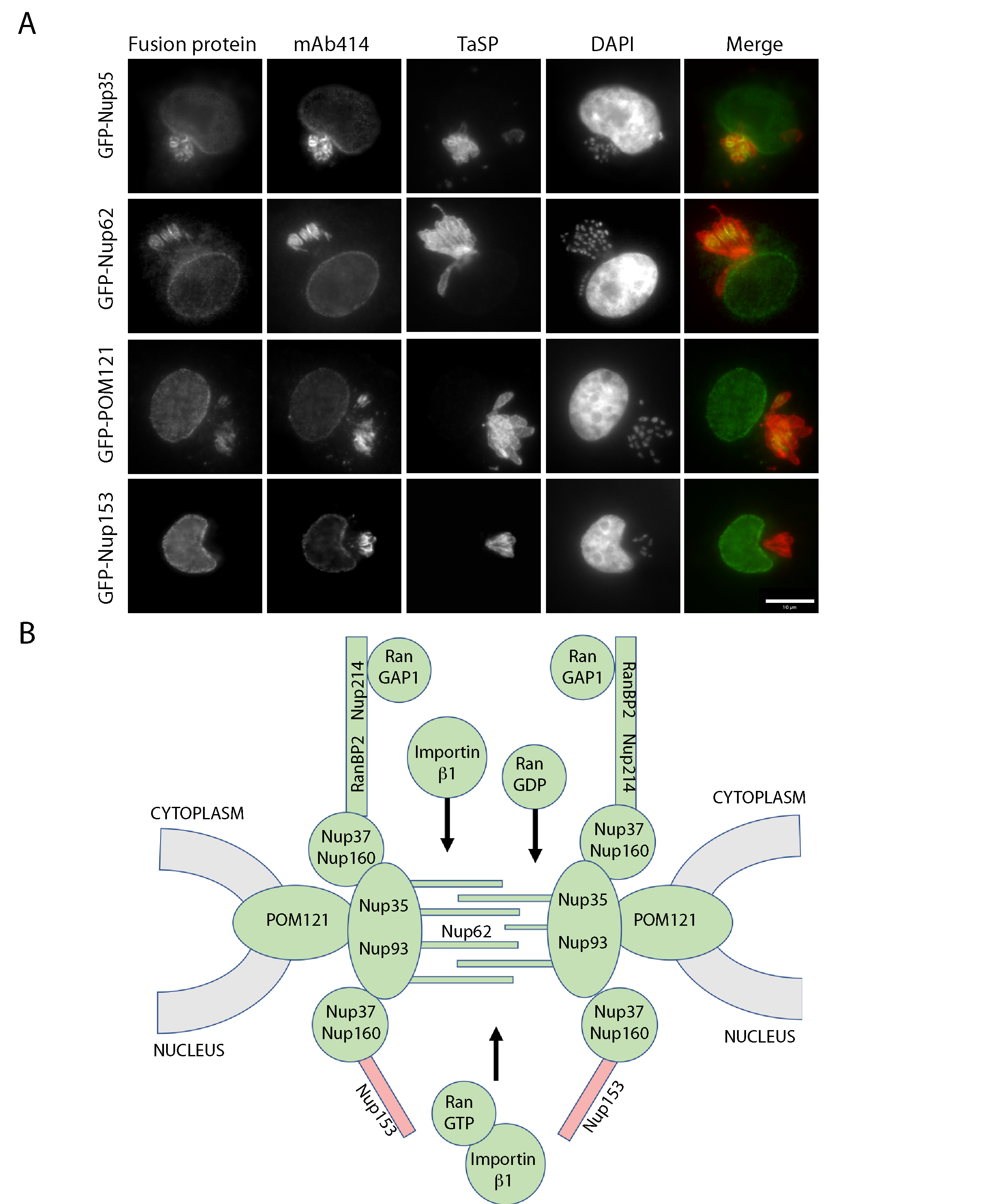

Supplement: FIG S2 [file mSphere.00709-19-sf002.tif]

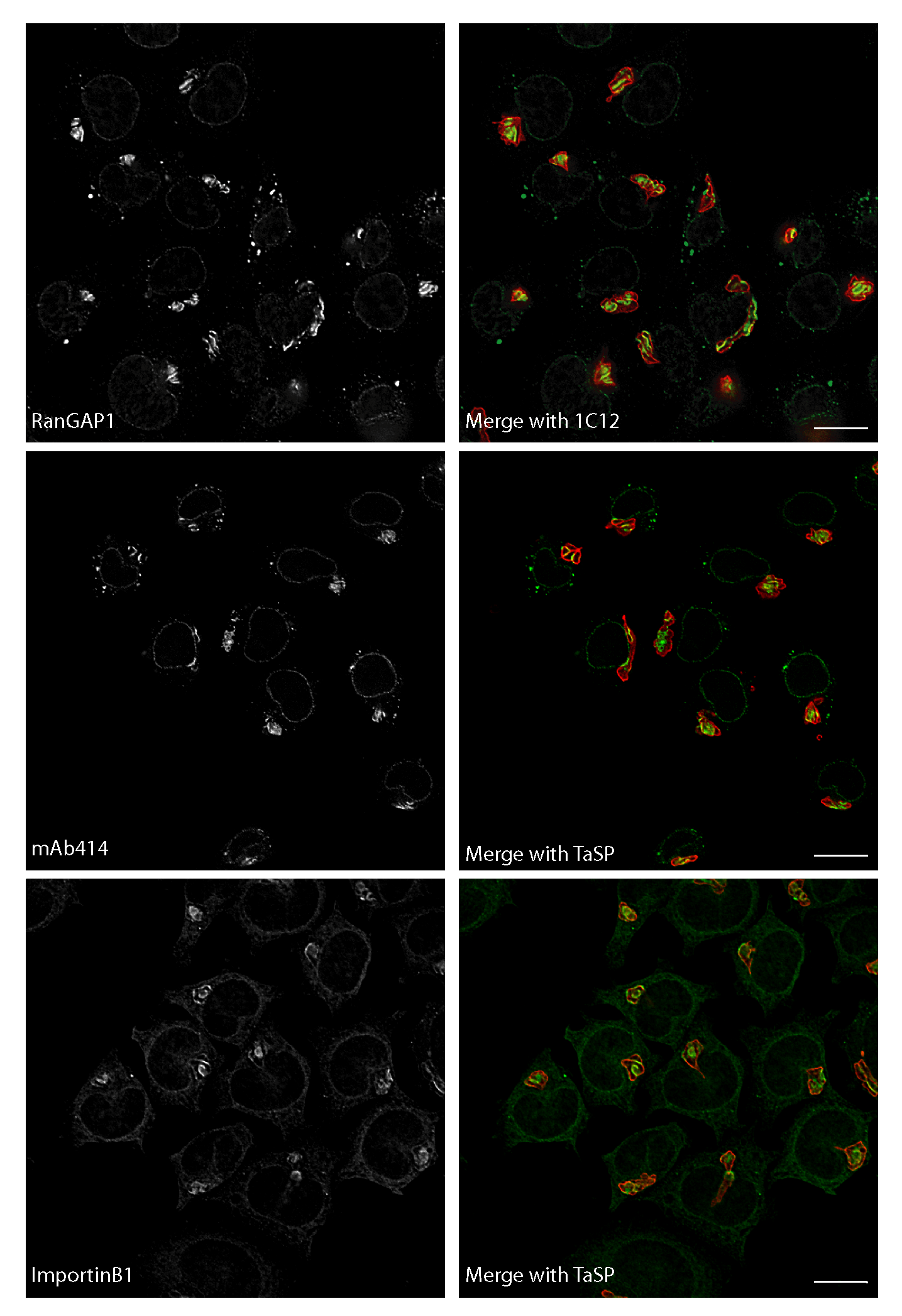

Supplement: FIG S3 [file mSphere.00709-19-sf003.tif]

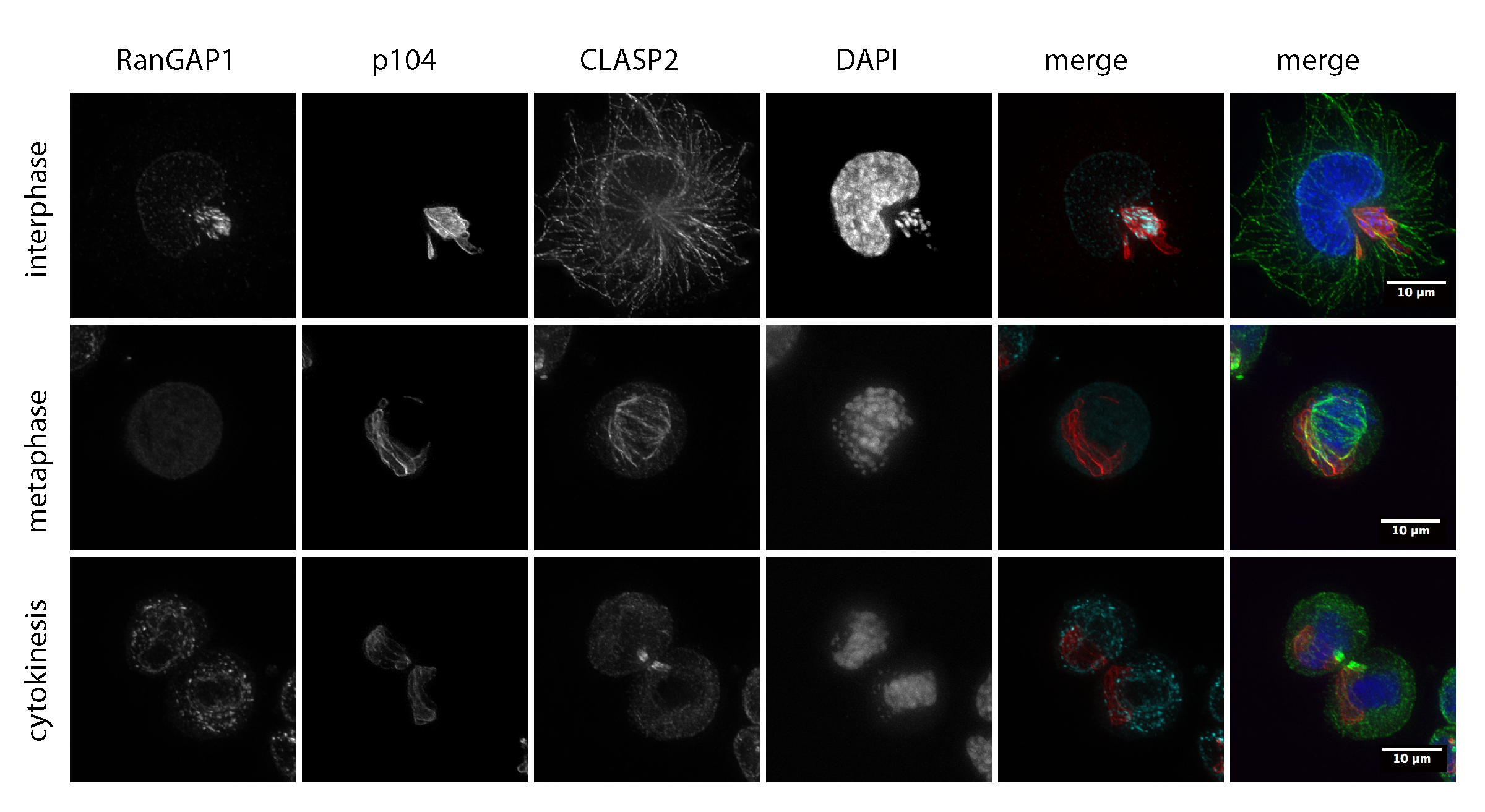

Supplement: FIG S4 [file mSphere.00709-19-sf004.tif]

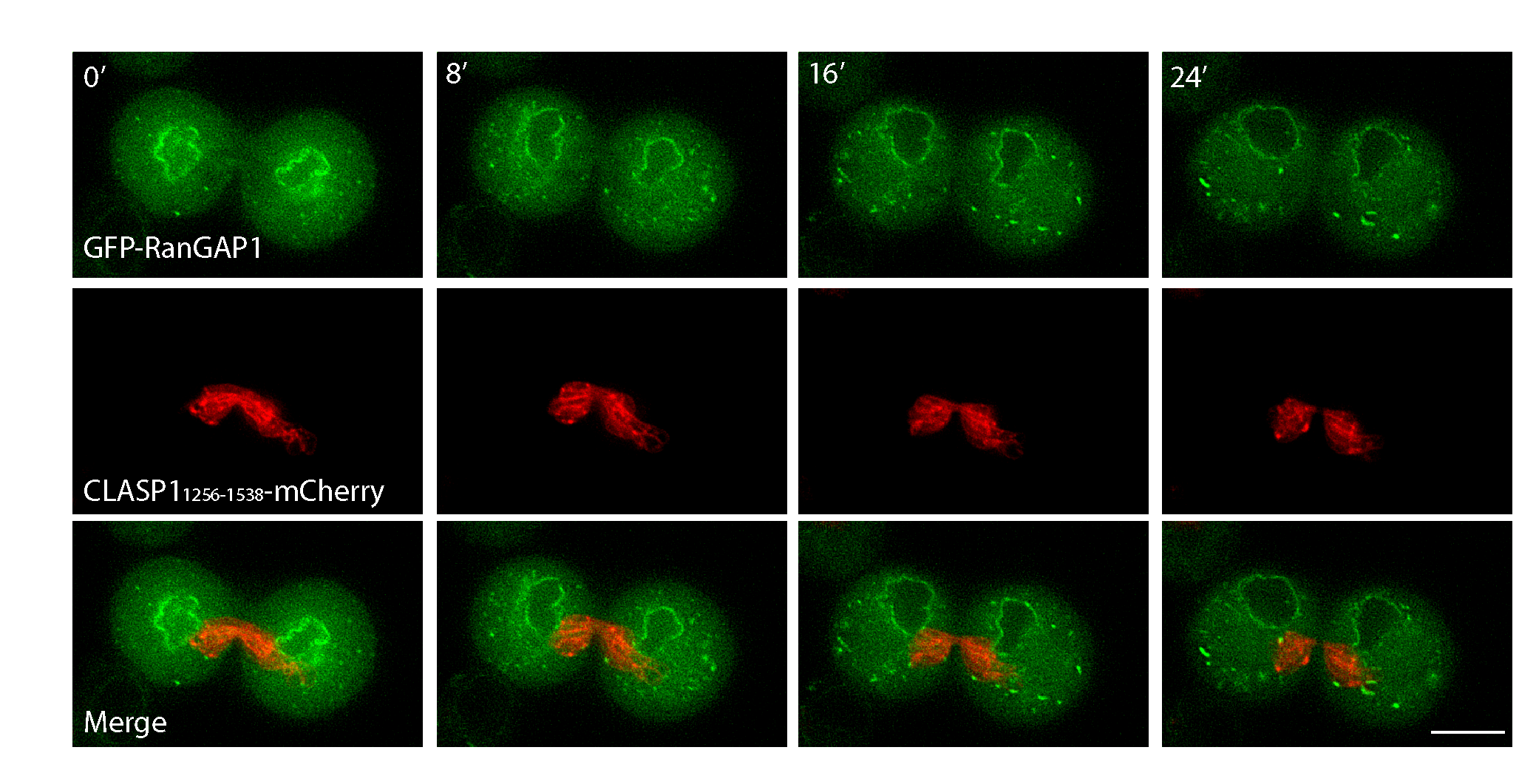

Supplement: FIG S5 [file mSphere.00709-19-sf005.tif]

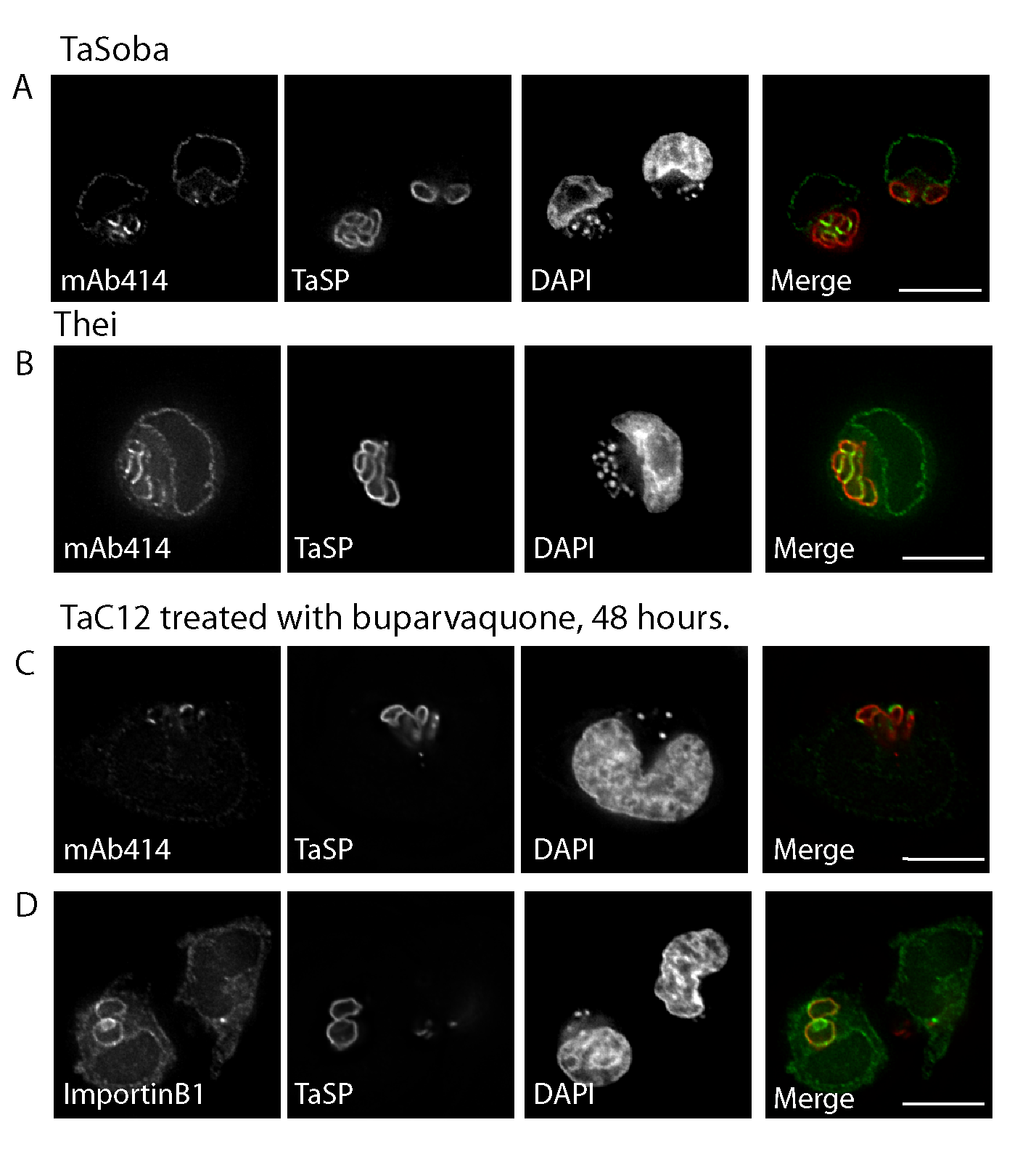

Supplement: FIG S6 [file mSphere.00709-19-sf006.tif]
